# Imaging of Group B Streptococcus infection in pyonephrosis: a case report^☆^

**DOI:** 10.1016/j.radcr.2022.06.014

**Published:** 2022-07-10

**Authors:** Ryan Elmanar, M. Hidayat Surya Atmaja

**Affiliations:** a Radiology Resident, Radiology Department, Faculty of Medicine, Universitas Airlangga - Dr. Soetomo Academic General Hospital, Jalan Mayjen. Prof. Dr. Moestopo 47, Surabaya, East Java, 60131, Indonesia; b Abdominal Radiologist, Radiology Department, Faculty of Medicine, Universitas Airlangga - Dr. Soetomo Academic General Hospital, Jalan Mayjen. Prof. Dr. Moestopo 47, Surabaya, East Java, 60131, Indonesia

**Keywords:** Pyonephrosis, GBS, *Streptococcus agalactiae,* Imaging

## Abstract

*Introduction:* Pyonephrosis is hydronephrosis accompanied by a bacterial infection in the kidney, causing suppurative destruction of the renal parenchyma; this condition is an emergency and usually associated with stones or chronic urinary tract infections. Urinalysis is typically inaccurate for establishing the diagnosis, as bacteriuria may not manifest due to ureteral obstruction. *Case report:* We reported a 55-year-old male patient with flanks pain and an account of stone expulsion. Based on history taking, physical examination, radiology examinations, and percutaneous nephrotomy, we concluded a diagnosis of pyonephrosis causing by *Streptococcus agalactiae* as known as Group B Streptococcus. *Discussion:* While both US and CT scan guided the early diagnosis, CT was more accurate as it is able to capture the renal function and the underlying cause of obstruction. Pyonephrosis was described as having a pus collection in the pelvicalyceal system, cortex thinning, and the appearance of stones. *Conclusion:* Pyonephrosis is a rare emergency, and many clinicians find it challenging to recognize since the presentations are frequently nonspecific. In order to prevent renal failure and the spread of bacteremia that entails life-threatening urosepsis, acquiring imaging knowledge (sonography and CT) and other findings are indispensable in determining this entity.

## Introduction

Pyonephrosis is a kidney infection accompanied by urinary tract obstruction symptoms. This disease represents an emergency condition because it prompts damage to the renal parenchyma and bacteremia, commonly caused by chronic obstruction due to kidney stones in adults. The mortality rates of bacteremia and urosepsis are 25% and 50%, respectively, and 15% of patients present asymptomatically [1].

**Fig. 1:**
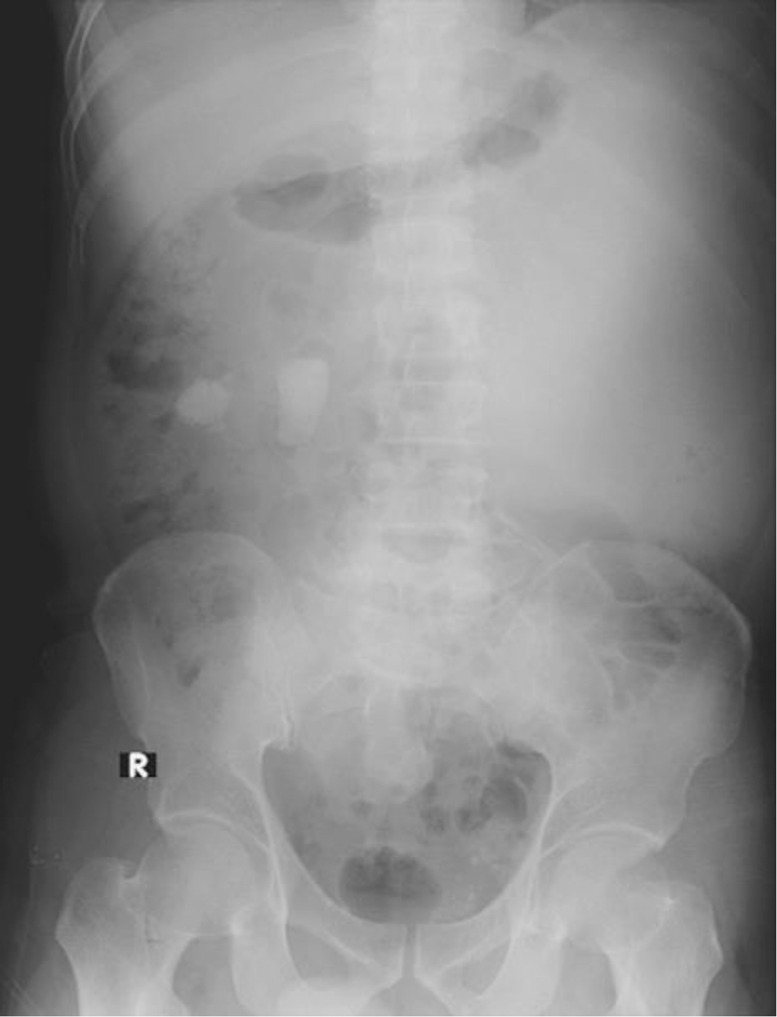
Abdominal X-ray showed ground-glass opacity at the left upper-lower quadrant abdomen accompanied by two oval-shaped opaque shadows at the level of 2nd – 3rd lumbar vertebrae.

**Fig. 2:**
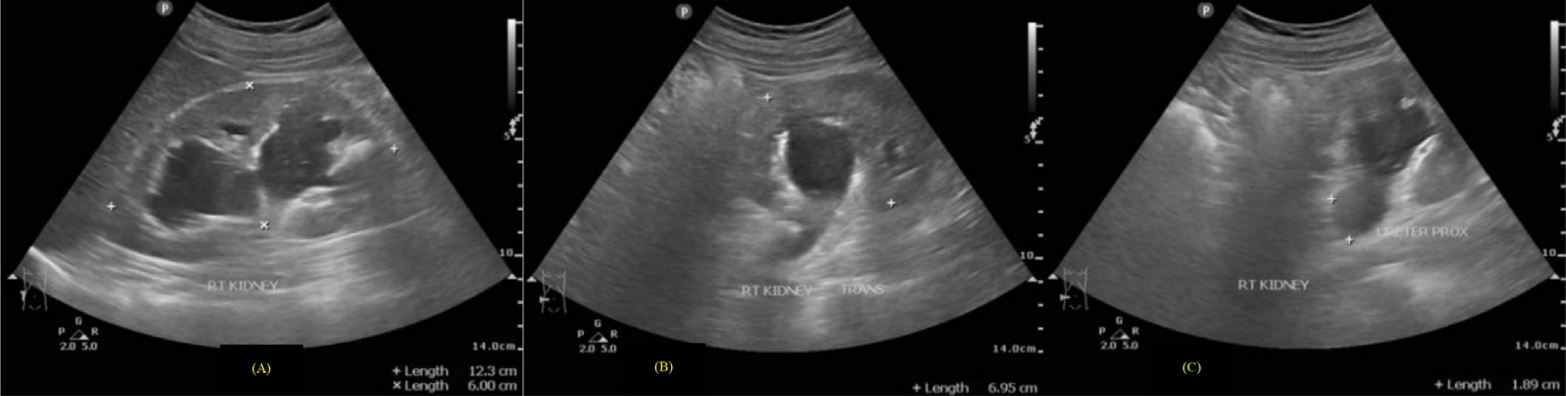
(A and B) Right pelvicalyceal ectasis with (C) proximal ureter dilatation.

Several examinations, such as urine analysis and urine culture, are mandatory to prove the presence of a bacterial infection in the urinary tract. The known *E. coli* was the most common microorganism (67.6%) of all uropathogens [2]. *Streptococcus agalactiae*, also known as Group B Streptococcus (GBS), made up only 1%-2% of all cases [3]. In contrast to other bacterial infections, patients with GBS infection had a significant increase in the probability of bacteremia, with more than 25% incidence of co-infection with staphylococcal species found [4].

**Fig. 3:**
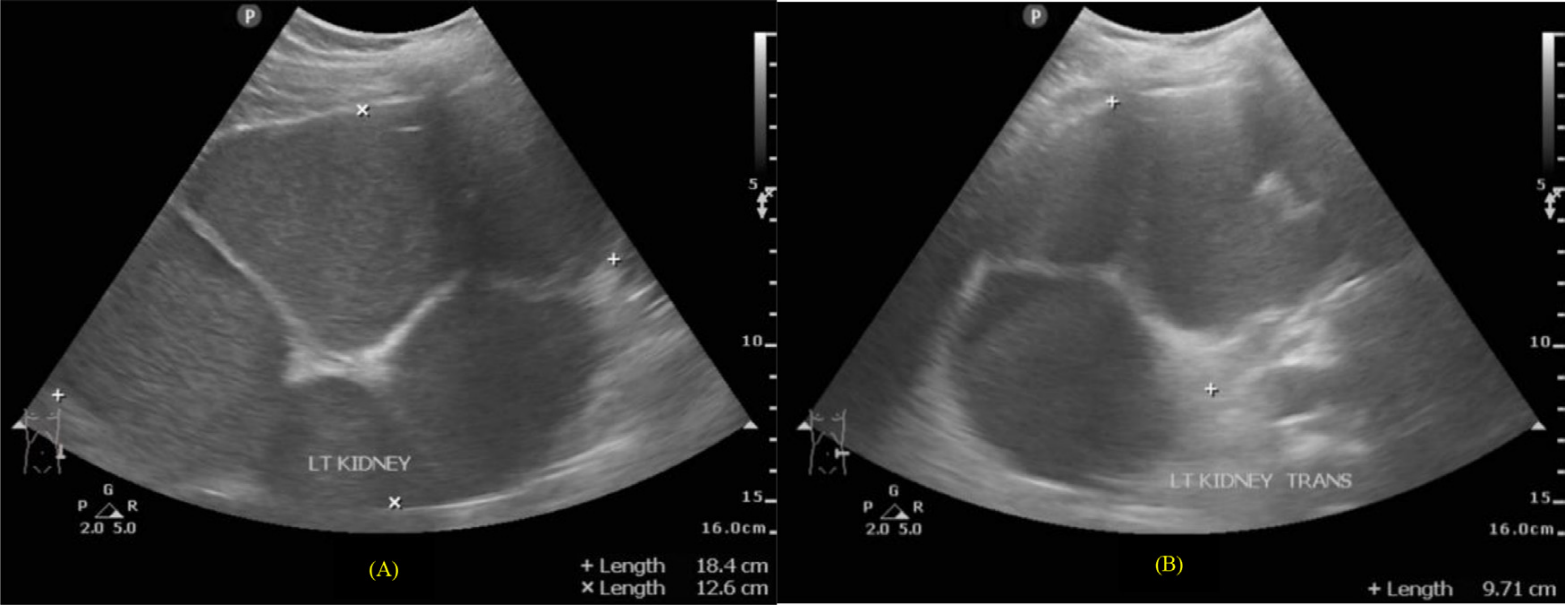
(A and B) sagittal and transverse views of the left kidney suggested severe hydronephrosis with layers of echogenic material and prominent fluid-fluid level at renal pelvic.

**Fig. 4:**
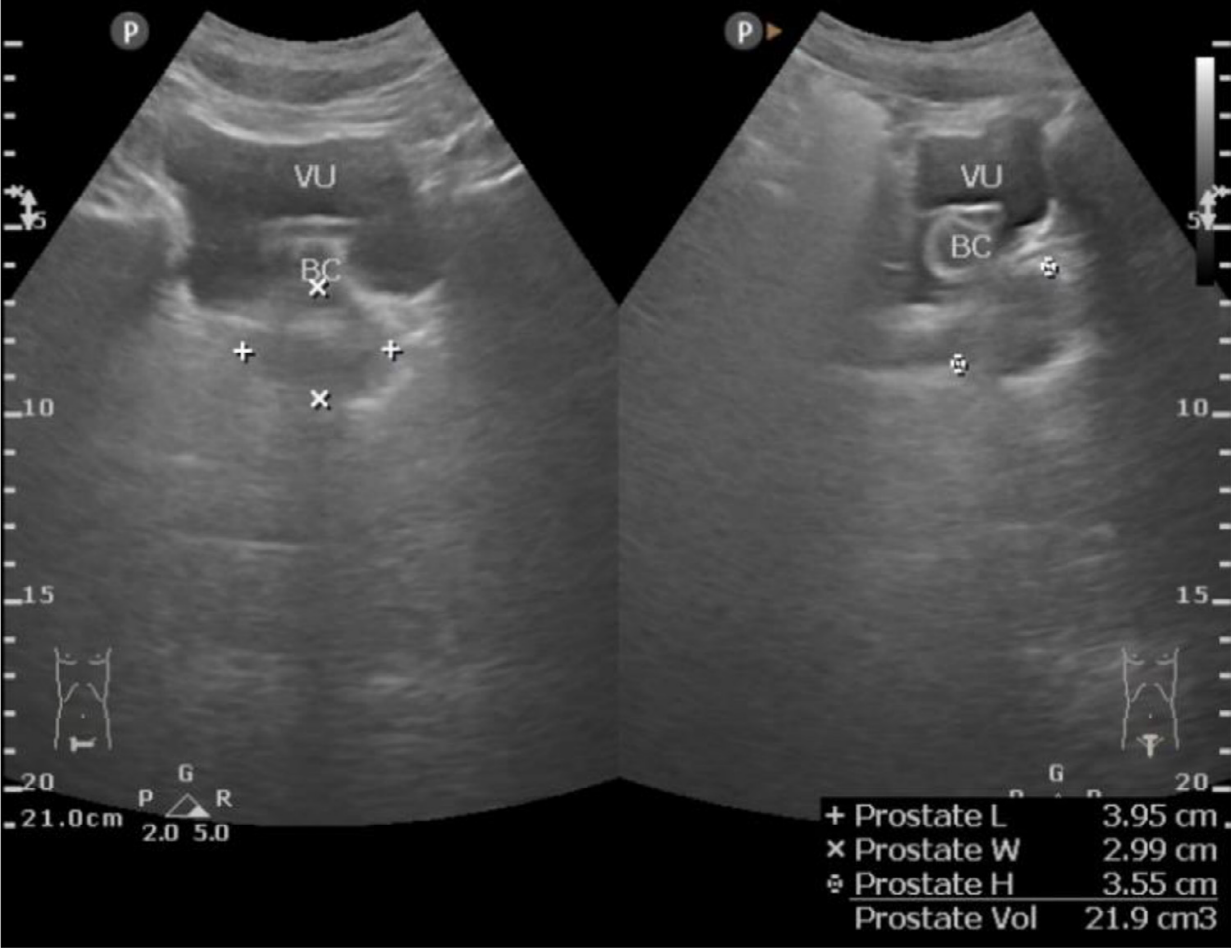
Normal prostate with a urinary catheter.

**Fig. 5:**
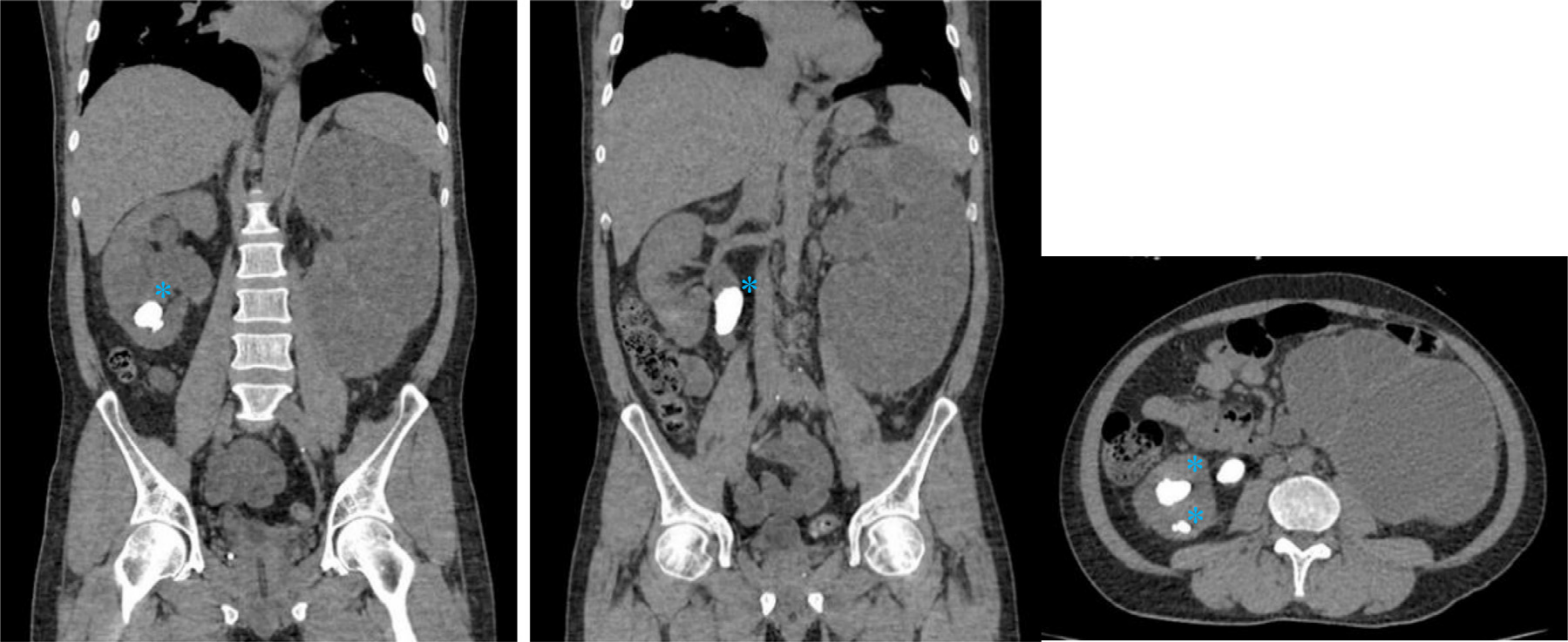
CT scan images depicted the stones (Asterix) at the proximal right ureter, causing dilatation of the structures above. Right hydronephrosis equivalent to 2nd – 3rd grade and right hydroureter were also visible.

**Fig. 6:**
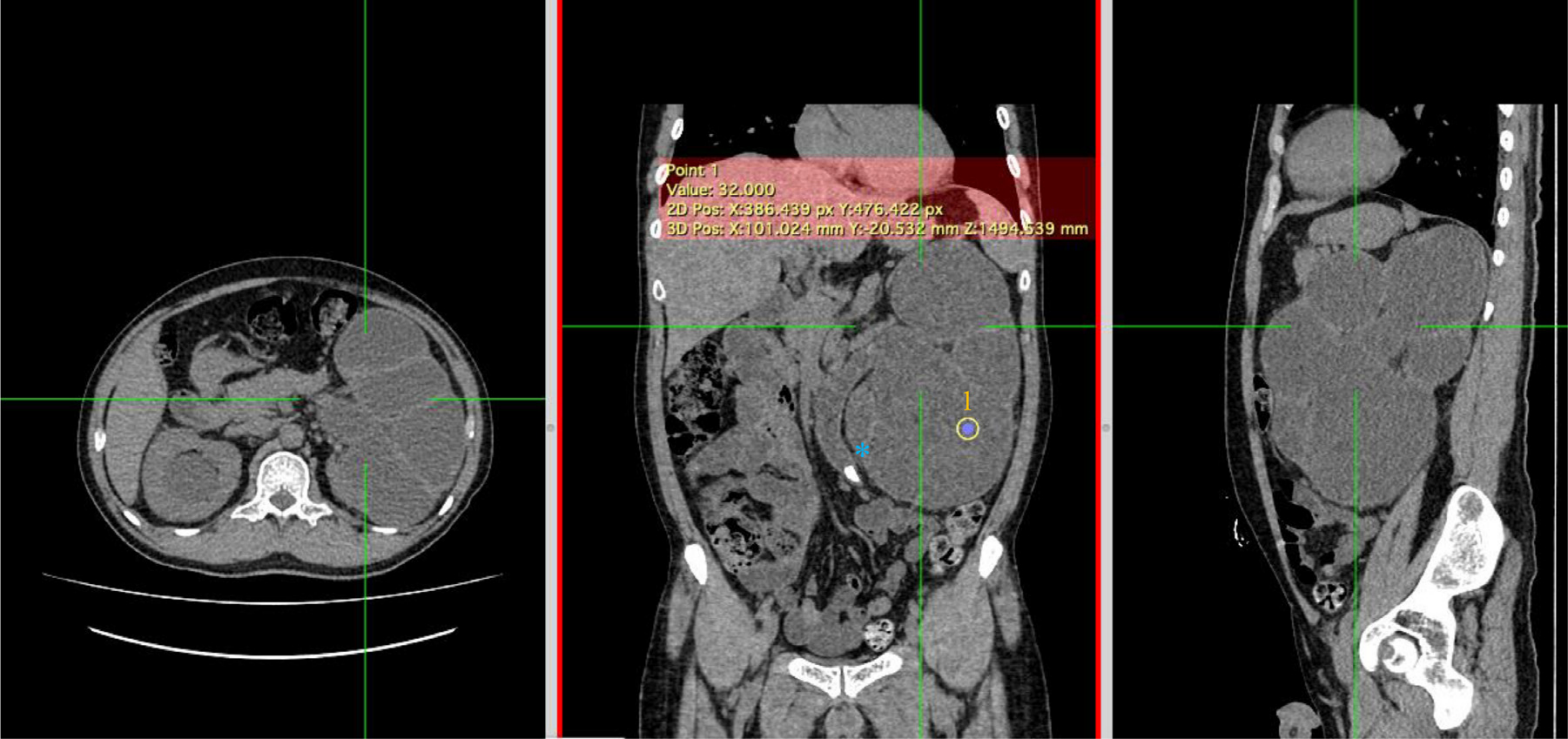
A renal stone (Asterix) in the left proximal ureter caused left proximal ureter dilatation. The presence of thick fluid in the left pelvicalyceal system and thinning of the renal cortex suggested left pyonephrosis with severe hydronephrosis and left hydroureter.

**Fig. 7:**
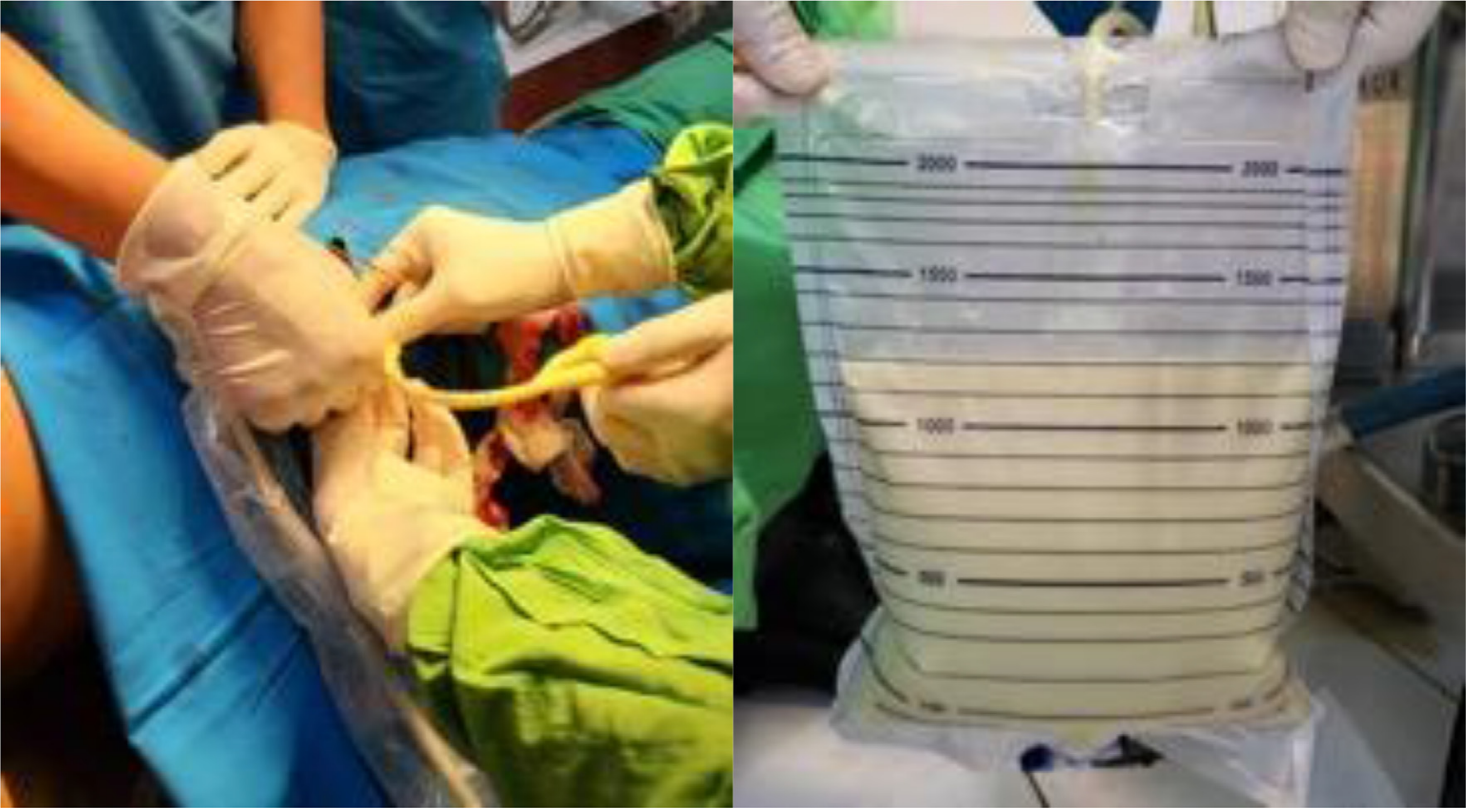
*Streptococcus agalactiae*, also known as Group B Streptococcus (GBS), was isolated after a percutaneous nephrostomy catheter was placed.

Patients with pyonephrosis primarily present with flank pain, tenderness, and a high fever, frequently associated with a history of renal stone, recurrent infection, or surgery. Additional complaints have included fever and abdominal discomfort on many occasions [5].

When the lumen is filled with purulent material, pyonephrosis with severe obstruction can be difficult to detect because bacteremia tests are generally negative; therefore, timely imaging is required to determine the best diagnosis. According to literature, ultrasound (US) can show internal mobile collecting system debris (with or without a fluid-debris level) and renal stone, as well as dilated pelvicalyceal system and echogenic debris, which is the most common reliable sign of pyonephrosis [1,6].

An abdominal CT scan plays a vital role in depicting pyonephrosis. It displays the hydronephrotic component, which is characterized by the presence of pus and an immoderate fluid attenuation in the collecting system. We can also clearly appreciate the collecting system and renal dilatation. Furthermore, MRI provides little additional information over CT imaging; however, it may be preferred to avoid intravenous contrast medium administration in several cases [6].

Finally, a pus culture is a gold standard for infectious agent diagnosis. Once the diagnosis of pyonephrosis is established, an adequate antibiotic must be administered promptly, followed by drainage of the infected pelvis [5].

## Case report

A 53 years old man presented to our center with right and left flank pain; a history of stone expulsion while urinating was also recorded several times measuring up to ± 1 × 0.2 cm, with no abdominal pain nor fever. A left flank mass was palpable during the physical examination and cystic mass pain. Urinalysis showed elevated ureic and creatinine levels, along with hematuria and pyuria.

Afterward, an abdominal X-ray was conducted, picturing ground-glass opacity at the left upper-lower quadrant abdomen and 2 oval-shaped opaque shadows at the right side at the second to third lumbar vertebrae level. The subsequent sonography revealed moderate right hydronephrosis and hydroureter and severe left hydronephrosis with echogenic debris and undefined cortex border. Abdominal CT showed thick fluid (13-38 HU) filling the left pelvicalyceal system, suggesting pyonephrosis with severe left hydronephrosis, moderate right hydronephrosis, bilateral hydroureter, and 2 stones in the lower pole of the right renal measuring around ± 1.6 × 1.8 × 2.3 cm and 1.5 × 1.1 × 1.4 cm, and a stone in the proximal left ureter sizing ± 1.6 × 3.6 × 1.9 cm.

Percutaneous nephrostomy catheter insertion guided by US for abscess drainage in left renal parenchyma produced ± 1200 mL of putrid exudate. Pus sample was cultured, resulting in GBS as the bacterial pathogen; therefore, an antibiotic was administered. Lithotripsy was performed several days onward, discovering ureterolithiasis and nephrolithiasis composed of phosphate, ammonium, calcium, and oxalate based on the stone analysis.

## Discussion

According to literature, pyonephrosis commonly occurs from acute or chronic obstruction secondary to a kidney stone [6]. In this case, the patient's age and history of several renal stones and decreased renal function predisposed the pyonephrosis and caused progressive damage to the renal parenchyma. The presence of multiple stones resulted in stagnation of urine, increasing the chance of infection [7].

It is known that bacterial infection can lead to the development of struvite stones. However, according to other reports, bacteria produce citrate lyase causing a fall in urine citrate levels, leading to supersaturated urine and crystal formation, and eventually, increased calcium oxalate deposition [8].

US and CT imaging aid in the early detection of pyonephrosis; however, CT is more precise than US as it identifies renal function, causes of obstruction and abdominal pathologies such as hydronephrosis [9]. Moreover, CT-based Hounsfield unit (HU) measurement could be used as a predictor for sepsis [10].

In our case, the plain radiograph pictured the ground-glass opacity at the left upper-lower quadrant of the abdomen. However, the abdominal x-ray was unable to distinguish pyonephrosis clearly. Therefore, following the literature, the US showed severe obstruction with pelvicalyceal system dilatation and echogenic material, suggesting pyonephrosis. The cause of the obstruction and dilatation can be determined using CT imaging. In the case study, pyonephrosis was manifested by thick fluid filling the pelvicalyceal system, alongside renal cortex thinning and renal stone in the left proximal ureter.

Lastly, a percutaneous nephrostomy catheter was inserted, followed by a pus culture resulting in GBS. Percutaneous nephrostomy is necessary for antibiotics to penetrate the renal parenchyma, which was given upon admission. Several days later, the definitive surgery for renal stones was performed with a satisfying result and no postoperative complications (Fig. 7).

GBS' role as an uropathogen remains unclear. There is no difference in clinical manifestations between the upper or lower urinary tract infection (UTI) [11]. Symptoms of GBS in UTI comprised of asymptomatic bacteriuria, cystitis, pyelonephritis, urethritis, and urosepsis. Nevertheless, only a minor percentage of patients are considered symptomless [12].

According to literature, most cases of GBS infection in adults had at least one risk factor, including cirrhosis, diabetes, stroke, breast cancer, decubitus ulcer or neurogenic bladder [13]. In another study, a prior history of UTI, women, and advanced age were also significant risk factors [12]. Nonetheless, there is no evidence of the chronic conditions mentioned above in our case. An abnormality of urinary flow and kidney stones also contributed to the incidence of UTI due to GBS, as stipulated by another report [11].

## Conclusion

This manuscript described a new case associated with GBS infection; pyonephrosis is a rare emergency case which should be diagnosed and treated promptly. Radiology imaging aids in proper diagnosis and provides more valuable information for further treatment, namely guiding insertion of drainage. Furthermore, apart from being used as a diagnostic tool, radiology could contribute as a predictor of serious complications.

## Patient consent statement

Written consent has been obtained from the patient as no identifiable patient data were included in this case report.

## Ethics committee approval

This study has met the ethical principle and received approval from the Research Ethics Committee of Dr Soetomo General Hospital, Surabaya.

## References

[1] Tublin M, Levine D, Thurston W, Wilson SR., Rumack CM, Levine D (2018). Diagnostic Ultrasound.

[2] Magliano E, Grazioli V, Deflorio L, Leuci AI, Mattina R, Romano P (2012). Gender and age-dependent etiology of community-acquired urinary tract infections. Sci World J.

[3] Mohanty S, Purohit G, Rath S, Seth RK, Mohanty RR. (2021). Urinary tract infection due to Group B Streptococcus: a case series from Eastern India. Clin Case Rep.

[4] Farley MM, Harvey RC, Stull T, Smith JD (1993). A population-based assessment of invasive disease due to Group B Streptococcus in nonpregnant adults. N Engl J Med.

[5] Cooper KL, Badalato GM, Rutman MP. (2021). Campbell-Walsh Urology.

[6] Kavanagh RG, Maher MM, O'Connor OJ, Adam A, Dixon AK, Gillard JH, Schaefer-Prokop CM (2021). Grainger & Allison's Diagnostic Radiology.

[7] Patodia M, Goel A, Singh V, Singh BP, Sinha RJ, Kumar M (2017). Are there any predictors of pyonephrosis in patients with renal calculus disease?. Urolithiasis.

[8] Schwaderer AL, Wolfe AJ. (2017). The association between bacteria and urinary stones. Ann Transl Med.

[9] Erol A, Çoban S, Tekin A. (2014). A giant case of pyonephrosis resulting from nephrolithiasis. Case Rep Urol.

[10] Boeri L, Fulgheri I, Palmisano F, Lievore E, Lorusso V, Ripa F (2020). Hounsfield unit attenuation value can differentiate pyonephrosis from hydronephrosis and predict septic complications in patients with obstructive uropathy. Sci Rep.

[11] Muñoz P, Coque T, Rodriguez Créixems M, Bernaldo De Quirós JCL, Moreno S, Bouza E (1992). Group B Streptococcus: a cause of urinary tract infection in nonpregnant adults. Clin Infect Dis.

[12] Ulett KB, Benjamin WH, Zhuo F, Xiao M, Kong F, Gilbert GL (2013). Diversity of group B streptococcus serotypes causing urinary tract infection in adults (Journal of Clinical Microbiology (2009) 47:7 (2055-2060)). J Clin Microbiol.

[13] Jackson LA, Hilsdon R, Farley MM, Harrison LH, Reingold AL, Plikaytis BD (1995). Risk factors for group B streptococcal disease in adults. Ann Intern Med.

